# Extra-pituitary midline structural abnormalities associated with ectopic posterior pituitary detected on a new rapid MRI protocol (FAST1.2)

**DOI:** 10.20945/2359-3997000000505

**Published:** 2022-08-04

**Authors:** Arthur Lyra, Daniel de Faria Guimarães, Altino Sá Meira, Guilherme Vieira Peixoto, Tatiane Sousa e Silva, Carlos Alberto Longui, Cristiane Kochi, Antônio José da Rocha

**Affiliations:** 1 Irmandade da Santa Casa de Misericórdia de São Paulo Unidade de Endocrinologia Pediátrica São Paulo SP Brasil Unidade de Endocrinologia Pediátrica, Irmandade da Santa Casa de Misericórdia de São Paulo, São Paulo, SP, Brasil; 2 Irmandade da Santa Casa de Misericórdia de São Paulo Departamento de Radiologia São Paulo SP Brasil Departamento de Radiologia, Irmandade da Santa Casa de Misericórdia de São Paulo, São Paulo, SP, Brasil; 3 Santa Casa de São Paulo Faculdade de Ciências Médicas São Paulo SP Brasil Faculdade de Ciências Médicas, Santa Casa de São Paulo, São Paulo, SP, Brasil

**Keywords:** Pituitary gland, pituitary diseases, posterior pituitary disease, growth hormone deficiency, panhypopituitarism

## Abstract

**Objective:**

Test if the MRI FAST1.2 protocol can detect extra-pituitary midline structural brain abnormalities in patients with ectopic posterior pituitary (EPP), and highlighting their radiological-laboratory correlations.

**Subjects and methods:**

Cross-sectional study of patients with EPP and control group. All individuals were submitted to FAST1.2, which combines the FAST1 protocol developed by our group with 3D T2DRIVE imaging.

**Results:**

We evaluated 36 individuals with EPP and 78 as control group. Pituitary stalk (PS) was identified in 7/36 patients in EPP group by FAST1, and in 24/36 patients in FAST1.2 (p < 0.001). FAST1 failed to detect PS in one individual in the control group, while the FAST1.2 defined the PS in all individuals. In EPP group, eleven had interhypothalamic adhesion (IHA), three septo-optic dysplasia, and one cerebellar malformation. We didn’t observe higher frequency of panhypopituitarism or developmental delay in patients with IHA. In control group, three had pars intermedia cysts, one hydrocephalus, and one hypothalamic hamartoma.

**Conclusions:**

FAST1.2 allows confident recognition of midline structural abnormalities, including the pituitary stalk and IHA, thereby making MRI acquisition faster and with no need for contrast administration. IHA could be associated with defects in neuronal migration, as occur in patients with EPP, with no clinical significance.

## INTRODUCTION

Ectopic posterior pituitary (EPP) is a congenital malformation of the hypothalamic-pituitary region resulting in various clinical outcomes from isolated growth hormone deficiency (GHD) to a combination of multiple pituitary deficiencies, known as panhypopituitarism ( 1 , 2 ). EPP is frequently associated with the thinning or absence of the pituitary stalk and/or with moderate to severe hypoplasia of the adenohypophysis, a triad also known as pituitary stalk interruption syndrome (PSIS). In this syndrome, which has an estimated incidence of 0.5/1,000,000 live births, clinical presentation tends to be more severe, with concomitant multiple pituitary hormone deficiencies ( 3 ). Magnetic resonance imaging (MRI) is the standard investigation method, depicting the anatomical abnormalities of the pituitary gland and hypothalamus. EPP is detected by MRI as a hyperintense T1 signal, corresponding to neurophysin II, usually located at the level of the infundibular recess of the third ventricle (median eminence), but it can be found anywhere along the infundibular axis ( 4 ). Additional structural brain abnormalities may be present in these individuals, such as the absence of a pituitary stalk, periventricular nodular heterotopia, bilateral perisylvian polymicrogyria, cerebellar vermis atrophy, cerebellar dysgenesis, partial agenesis of the corpus callosum, Chiari malformations, agenesis of the septum pellucidum, aqueductal stenosis, and optic nerve hypoplasia ( 1 , 2 ).

Our group first described the FAST1 MRI protocol in 2004, an MRI method that uses a T1-weighted sagittal sequence without the use of contrast or anesthesia, and lasting only 3.25 minutes. In that study, FAST1 showed complete agreement with the classic protocols with intravenous gadolinium administration. Therefore, this protocol could be employed in large-scale investigations of patients with short stature ( 5 ). However, the T1 MRI sagittal sequence pattern is not able to perfectly detect small changes in the pituitary stalk. To do this, it is necessary to perform a T1 weighted sequence with gadolinium administration or the T2-weighted sequences (T2 weighted images with very thin slices preferably using 3D acquisition sequences, which are named differently according to the manufacturer (CISS, DRIVE, or FIESTA) ( 6 , 7 ). Therefore, we started assessing patients with EPP using the FAST1.2 protocol, which consists of FAST1 with the addition of T2 DRIVE acquisition ( 6 , 7 ). This new FAST1.2 protocol provides detailed information on the anatomy of the suprasellar compartment, as a result of differences between the cerebrospinal fluid and the adjacent parenchymal structures. The images allow an accurate assessment of the pituitary stalk while avoiding the use of intravenous contrast administration and therefore maintaining the major characteristic of the FAST1 protocol, namely its short duration protocol, with the new method taking less than 7 minutes when using a 1.5 Tesla MR scanner.

Our main goal was to scrutinize midline structural abnormalities in patients with EPP, with a secondary objective of characterizing the clinical-radiological correlations with the degree of pituitary deficiency.

## SUBJECTS AND METHODS

This was a cross-sectional study, which included individuals of both genders followed up at a Pediatric Endocrinology Unit of a Tertiary Hospital, with a previous MRI diagnosis of short stature due to hypothalamic-pituitary hormone deficiency secondary to EPP. GH deficiency was diagnosed by a GH peak response of < 5 ng/mL in clonidine and insulin stimulation tests. Central adrenal insufficiency was defined as 08:00 a.m. cortisol values lower than 5 mcg/dL and low/normal levels of adrenocorticotropic hormone (ACTH). Central hypothyroidism was defined as reduced free T4 values, associated with low or normal thyroid-stimulating hormone (TSH) values. Hypogonadotropic hypogonadism was diagnosed as the absence of puberty in girls after 13 years and boys after 14 years of age, associated with low gonadotropins, estradiol, or total testosterone.

The control group consisted of individuals who underwent MRI concerning other differential diagnoses for short stature, such as idiopathic short stature (ISS), including constitutional delay growth and puberty, and patients with central precocious puberty (CPP). The exclusion criteria were patients with hypopituitarism caused by tumors or cerebral brain injury, in addition to those with an orthodontic appliance or hearing prosthesis that could have resulted in artifacts limiting imaging interpretation. The Ethics Committee approved the study protocol (CAAE: 85543918.0.0000.5479). Written informed consent was obtained from subjects and/or their guardians, and the study followed the principles of the Declaration of Helsinki. The inclusion period was from March 2018 to December 2019.

The MRI of the hypothalamic-pituitary region was performed at the Diagnostic Imaging Service, using a Philips Gyroscan 1.5T and Philips Achieva 1.5T MR scanner. FAST1, as previously described, lasting 3.25 minutes was performed without intravenous contrast administration and comprised 12 slices with a thickness of 2 mm and a gap of 0.2 mm ( 5 ). Additional sagittal T2 DRIVE used TR/TE 1500/250 to obtain 40 consecutive slices in a 3D acquisition, with a thickness of 0.6 mm, lasting 3.37 minutes. All patients were subject to fluid restriction for at least 6 hours for adequate visualization of the neurohypophysis hyperintense signal. The whole imaging acquisition in FAST1.2 lasted about 7 minutes.

A neuroradiologist (AJR) with more than 20 years of experience analyzed the images. In both groups, the structural images were scrutinized to define the location of the posterior pituitary lobe, the presence or interruption of the pituitary stalk, and to actively search for midline regional brain abnormalities, including gyri anatomy, corpus callosum dysgenesis, ventricular morphology, and optic nerve abnormalities, among others possible structural abnormalities visible using the 3D acquisition in this FAST1.2 protocol.

Statistical analysis was performed using the program SigmaStat 3.5 for Windows (SPSS, Point Richmond, CA, USA). We used a Z-test to compare the proportion of findings between groups. A p-value of <0.05 and a 95% confidence interval were considered statistically significant. The study data were collected and managed using REDCap (Research Electronic Data Capture) electronic data capture tool hosted at Santa Casa SP, School of Medical Sciences. REDCap is a secure, web-based software platform providing validated data capture and audit trails.

## RESULTS

**Figure 1:**
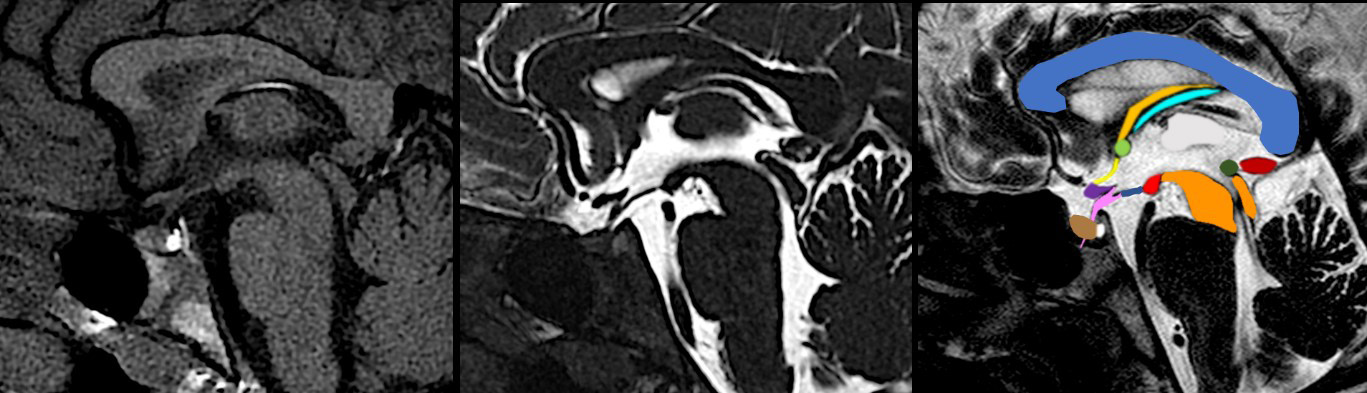
FAST1.2 in a normal structural appearance of the hypothalamic-pituitary axis. On the left, FAST1 imaging with a normal pituitary stalk (black arrow), normal pituitary lobes, and eutopic pituitary posterior lobe inside the sella turcica (white arrow). In The middle, Additional T2 DRIVE image allowed us to define the W appearance normal structures, compare to colored image. Except of the mammillary bodies (red) and tuber cinereum (dark blue), the hypothalami are not visible at midline. The corpus callosum (light blue), fornix (dark yellow), choroid plexus of III ventricle (water green), thalamus (grey), choroid plexus midbrain (dark orange), posterior commissure (dark green), pineal gland (dark red), lamina terminalis (yellow), anterior commissure (light green), optic chiasm (dark purple), pituitary stalk (pink), adenohypophysis (brown), and neurohypophysis (white) are represented.

**Figure 2:**
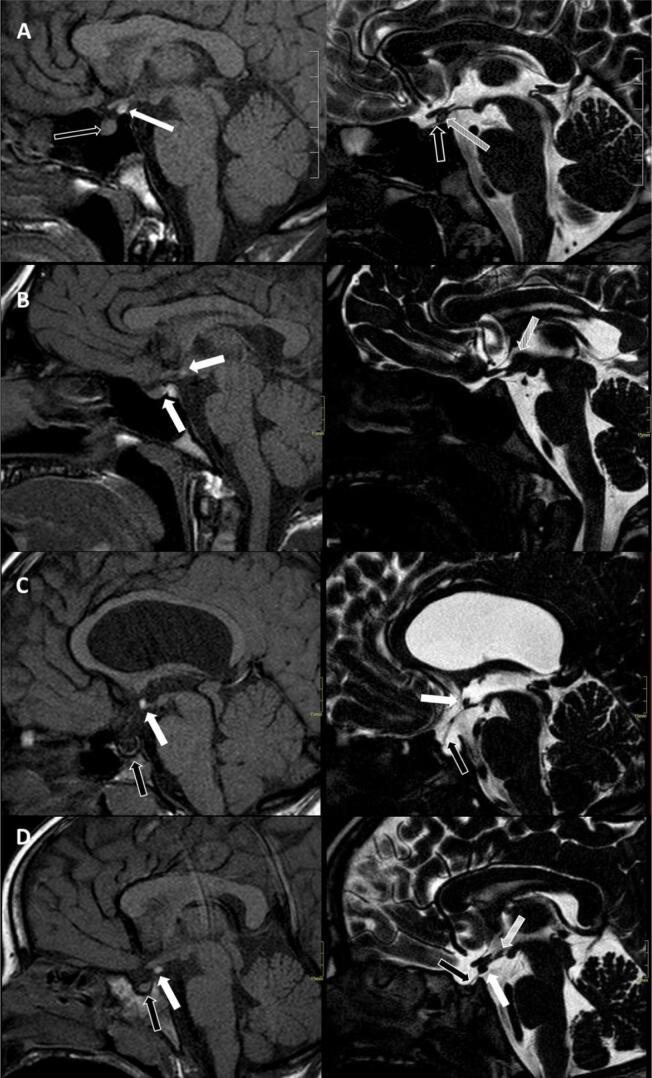
Evaluating the superimposed acquisition of FAST1.2 protocol in 4 patients ( **A, B, C, D** ), on the left FAST1 and on the right T2DRIVE images. **A:** FAST1 imaging with EPP (white arrow), pituitary anterior lobe (black arrow), and absence of the pituitary stalk. T2 DRIVE protocol with pituitary posterior lobe impression (striped arrow) along the proximal part of pituitary stalk (black arrow). **B:** FAST1 shows two posterior pituitary lobes, one inside the sella turca and another along the pituitary stalk (white arrows). T2 DRIVE protocol demonstrates pituitary stalk and the presence of interhypothalamic adhesion above the tuber cinereum and mamillary bodies (striped arrow). **C:** FAST1 imaging with septo-optic dysplasia with complete hypothalamic fusion with a unified recess in the floor of the third ventricle recess, hypothalamic EPP (white arrow), and empty sella (black arrow). In T2DRIVE, EPP shadow (white arrow) inside the third ventricle and pituitary stalk clearly defined pituitary stalk (black arrow). **D:** FAST1 demonstrates median eminence EPP (white arrow) and normal anterior pituitary lobe (black arrow). T2DRIVE shows EPP shadow (white arrow), pituitary stalk (black arrow), and interhypothalamic adhesion adjacent to tuber cinereum (striped arrow), not visible on T1.

In this study, we evaluated 36 individuals with EPP (aged between 5 and 27 years, with a mean age of 15.7 years), 30 of whom were male. Figure 1 depicts a midline sagittal graphic normal representation of the hypothalamic-pituitary region. The position of the posterior pituitary lobe, the size of the anterior pituitary lobe, the presence of a pituitary stalk, and other changes in the hypothalamic-pituitary region are shown in Figure 2 . In the EPP group, all patients had GH deficiency (defined by GH peak response < 5 ng/mL in two stimulation tests – clonidine and insulin). Central hypothyroidism was diagnosed in 28 patients, defined as reduced free T4 values, associated with low/normal TSH values. Adrenal insufficiency was detected in 25 patients, with 8:00 a.m. cortisol values lower than 5 µg/dL and low/normal levels of ACTH. Finally, 23 individuals had hypogonadotropic hypogonadism, defined as the absence of puberty in girls after 13 years of age and boys after 14 years of age, associated with low gonadotropins, estradiol, or total testosterone. Fifteen patients had a diagnosis of variable developmental delay.

One patient had two lobes of the posterior pituitary, one inside the sella turcica and the other along the pituitary stalk ( Figure 2B ). In five patients, the posterior pituitary lobe was along the pituitary stalk ( Figure 2A ); in 28 patients it was located at the infundibular recess of the third ventricle ( Figure 2D ), and in two patients the EPP was hypothalamic ( Figure 2C ). The details of the extra-pituitary findings of these groups are described in Table 1 . The control group (n: 78) had a mean chronological age of 10.5 y (range: 5 to 19 years) and comprised 42 males and 36 females. The group consisted of 32 patients with central precocious puberty (CPP) and 46 with idiopathic short stature (ISS), all with normotopic posterior lobe in the evaluation. Table 1 summarizes the diagnoses and extra-pituitary findings in the control group. Figure 1 depicts a normal MRI found in one of the control group individuals.

**Table 1:** Extra-pituitary midline abnormalities detected on FAST1.2 in our series patients with EPP and control group.

Extra-pituitary findings	Number of patients with EPP	Number of patients in control group
Interhypothalamic adhesion	11	0
Septum pellucidum agenesia, partial agenesis of the corpus callosum, optic nerve hypoplasia	3	0
Left cerebellar hemisphere dysplasia	1	0
Pars intermedia cyst	0	3
Hydrocephalus	0	1
Hypothalamic hamartoma	0	1

The pituitary stalk was identified in 7/36 patients in the EPP group by FAST1 MRI, and in 24/36 patients by the FAST1.2 protocol (p < 0.001). FAST1 MRI failed to detect pituitary stalk in only one individual in the control group, while the FAST1.2 protocol defined the pituitary stalk in all individuals.

Concerning the clinical aspects in the EPP group, we found that 2/8 (25%) patients with isolated GHD had IHA and 9/28 (32%) had multiple pituitary hormone deficiencies (MPHD) and IHA (p = 0.961). We also notice de presence of 3/15 (20%) individuals with developmental delay and AIH and 7/24 (29%) with normal development and AIH (p = 0.794).

## DISCUSSION

Previous studies have reported various extra-pituitary cerebral anomalies in patients with EPP such as craniofacial dysmorphism, a central maxillary incisor tooth, septo-optic dysplasia (SOD), optic nerve hypoplasia, corpus callosal dysgenesis, cellular migration disorder, persistent cricopharyngeal canal, microcephaly, cerebellar atrophy, vermian dysplasia, Chiari malformation type-1, and ophthalmic abnormalities ( 3 , 6 , 8 ). It has been suggested that the coexistence of EPP with periventricular heterotopias occurs because both conditions share a common underlying causative mechanism of abnormal neuronal migration. Despite our protocol was only dedicated to the midline structures, pachygyria complex was confidently documented in three of our patients, associated with EPP, reinforcing that the theory of abnormal neuronal migration and cleavage anomaly is possibly enrolled in the pathogenesis of EPP ( 9 ).

Interhypothalamic adhesion (IHA) was the most frequently observed structural abnormality in our series of patients with documented extra-pituitary abnormalities (11/36). IHA was located adjacent to or above the tuber cinereum. It was not observed in any patient of the control group. To the best of our knowledge, this finding is not a common abnormality reported in patients with EPP, probably identified in this study as a result of T2 DRIVE resolution with very thin slices and contrast between cerebrospinal fluid (CSF) and hypothalamic structures.

IHA represents a gray matter-like linear band of tissue traversing the third ventricle anterior to the mammillary bodies, and is structurally similar, but anatomically distinct, from interthalamic adhesion. It has been incidentally observed through the routine acquisition of high-resolution T1 imaging ( 10 ). This subtle anomaly is primarily apparent on isotropic T1 imaging, it is under-recognized, especially because smaller IHAs are more likely to be asymptomatic. However, as high-resolution MRI techniques become more routinely used, IHAs are likely to be increasingly identified and should prompt detailed scrutiny for additional brain malformation in several different scenarios, including healthy subjects. Ahmed et al. described IHA in 57 patients who underwent MRI for neurological symptoms (such as seizures, delayed development, and trauma), but only four of them had SOD. Forty of the 57 patients had no clinical symptoms referable to their IHA. The authors concluded that IHA in patients without specific referable symptoms, and with few or no other structural abnormalities, could be incidental and of no clinical significance ( 10 ).

In the group with EPP, none of our patients with SOD had IHA. We could not find any difference between the presence of MPHD or developmental delay that could indicate a more severe phenotype associated with IHA. Our results reinforce the hypothesis that IHA might be a frequent structural abnormality associated with EPP but not detected using only T1 acquisition protocols, reinforcing the need for a high-resolution 3D acquisition to refine the structural analysis of midline structures, including to evaluate hypothalamus and pituitary gland.

It has previously been indicated that high-resolution imaging in all three planes (sagittal, coronal, and axial) is necessary to distinguish IHA from other hypothalamic disorders, such as hamartoma or glioma. Our proposed protocol (FAST1.2) is based on a 3D acquisition that allows IHA recognition based on its structural features by imaging, while at the same time reducing the time required for the examination and avoiding the need for gadolinium administration. The theoretical distinction of IHA from hamartoma or glioma will not represent a real difficulty, as these disorders are not commonly reported in association with EPP. Conversely, a round/ovoid lesion identified in a patient with confirmed EPP in the previously reported positions (crossing the third ventricle from one side to another of the hypothalamus), probably represented an IHA ( 10 , 11 ).

Although the underlying etiology of IHA is unclear, it may be the result of incomplete hypothalamic cleavage, failed apoptosis, or abnormal neuronal migration. Ahmed et al. pointed out the association between IHA and the gray matter heterotopia (GMH), suggesting that abnormal neuronal migration could play an important role in the etiology of this condition ( 10 ). Whitehead et al observed that accompanying structural abnormalities range from mild hippocampal under-rotation to severe malformations of brain development and midline anomalies are coexistent in most patients, supporting a type of form fruste holoprosencephaly. Some of the pathogenic variants associated with EPP were in genes responsible for neuronal proliferation and migration, such as *IFT172* , *SOX3* , and *LHX4* ( 8 ), suggesting that in the presence of EPP or other midline abnormalities, the IHA could be part of a Pleiades of structural abnormalities, potentially linked by an abnormal process of neuronal development or migration.

In conclusion, FAST1.2 MRI protocol was useful to investigate patients with short stature allowing more confident recognition of regional anatomy of the hypothalamic-pituitary region and depicting midline structural abnormalities on the T2 DRIVE imaging, precisely detecting the pituitary stalk, as well as IHA, with rapid MRI acquisition without intravenous gadolinium administration. Our results support the suspicion that interhypothalamic adhesion could be associated with defects in neuronal migration, as might occur in patients with EPP and that it could be of no clinical significance, as we did not observe a higher frequency MPHD or developmental delay in patients with IHA and EPP. Further studies are required to define the clinical correlations and genetic basis of the midline and also whole-brain structural abnormalities associated with EPP, clarifying the molecular mechanisms involved, and refining the appropriated scenarios to the extended MRI protocols.
